# “A Feeling of Safeness and Freedom”: The Promotion of Mental Health Recovery Through Co-Production in an Italian Community Organization

**DOI:** 10.1007/s10597-024-01279-2

**Published:** 2024-04-26

**Authors:** Antonella Guarino, Luca Negrogno, Christian Compare, Alessandra Madeo, Pamela Bolognini, Linda Degli Esposti, Michele Filippi, Francesca Lamberini, Martina Morrone, Matteo Masetti, Antonio Marco Serra, Cinzia Albanesi

**Affiliations:** 1 Dipartimento di Psicologia “Renzo Canestrari”, Alma Mater Studiorum Università di Bologna, Bologna, Italy; 2 Istituzione G.F. Minguzzi, Città Metropolitana di Bologna, Bologna, Italy; 3 3L’Arco – Corrispondenze per la recovery, Bologna, Italy

**Keywords:** Recovery, Mental health, Co-production, Community, Empowerment, Peer support workers

## Abstract

In mental health promotion, recovery is a process that leads to personal strengthening, control over crucial life decisions, and participation in communities through relevant professional, educational, or family social roles. Co-production, a key aspect of the recovery-oriented approach, emphasizes collaboration and active participation of people with mental health first-hand experience, family members, and citizens. Even though studies on co-production are limited and fragmented, there is evidence that co-production leads to positive outcomes, including improved well-being, empowerment, social connectedness, inclusion, and personal competencies. This study aimed to contribute to the limited literature on co-production in mental health by evaluating the co-production process in a non-profit mental health organization and its impact on empowerment processes and personal recovery outcomes. The research team adopted a collaborative approach and conducted qualitative research, including 13 individual semi-structured interviews and four focus groups. Results showed how the different dimensions of empowerment are promoted in and by the organization: (a) co-production processes supported empowered outcomes on an individual level, such as self-awareness; (b) the organization was perceived to promote empowering processes, such as a sense of safeness and protection; (c) co-production was a mean to build and maintain a network with mental health services that acknowledges the dignity and value of each subjectivity and promotes participation and recovery. Peer support workers were seen as facilitators of mental illness management, and the organization as a place for sharing mental health experiences and fostering individual recovery journeys.

## Introduction

Nearly one billion people worldwide live with a diagnosable mental disorder, and many do not have access to adequate care due to a lack of services, capacity, affordability, or stigma (WHO, 2013). In Italy, the COVID-19 pandemic has significantly impacted people’s mental health. Depression, anxiety, and stress have been reported as the most common mental health issues during the pandemic for various reasons, including social isolation, uncertainty, fear of illness, financial insecurity, and grief over losing loved ones loss (Cuomo et al., 2022).

Overall, the COVID-19 pandemic has highlighted the importance of mental health care and the need for innovative approaches to support people during times of crisis. As the Report on Mental Health (SISM, 2021) shows, across the Italian population the most rated diagnoses for male users are schizophrenia, personality disorders, substance abuse disorders, and mental retardation, while diagnoses of affective, neurotic, and depressive disorders are higher among cis-gender females. Specifically, the rate of female users affected by depression is almost double that of cis-gender males (40.4 per 10,000 inhabitants vs. 24.2 per 10,000 inhabitants). Considering the Italian system’s responsiveness to the care and treatment of mental illness, much still needs to be done, both in terms of training and continuity of care for people who are struggling with a mental disorder (Starace & Minguzzi, 2022).

WHO (2021, 2013) acknowledges that health systems are struggling to meet the needs of people with newly presenting as well as pre-existing mental health conditions, thus a *recovery-oriented paradigm* is suggested to focus on community-based treatments, rights protection, abuse, and poverty prevention, and address economic and social inequalities to transform mental health and reshape mental health systems and services. Indeed, a recovery-based approach includes an active role for individuals in their recovery journey, involving collaboration with all clinical and informal actors (WHO, 2013).

## Mental Health Recovery and Co-Production Processes

Mental health recovery is grounded in the experience of recovering from mental illness, not as a return to an initial state, but rather as a process of regaining one’s life and identity (Ornelas et al., 2019).

The *intervention-first approach*, based on community psychology principles (Sacchetto et al., 2022), posits that recovery as a personal process is only possible if individuals are involved in natural community environments and have concrete opportunities for participation (Ornelas et al., 2019). Recent reviews have gathered evidence on and described several participatory methods for involving people in mental health recovery. These methods include participatory action research, community-based action research, co-production, cooperative inquiry, and participatory appraisal. Despite being categorized under the umbrella term of participatory approach, these methods show few differences among them. The emphasis of these reviews lies in highlighting the commonalities, particularly the significance of promoting active participation and conducting collaborative research involving key stakeholders (Halvorsrud et al., 2021; Raanaas et al., 2020). A relevant method is the health co-inquiry, which relies on the full integration and participation of persons with chronic health conditions, caregivers, providers, and researchers and on mixing conventional and action research to foster translation of results into future practices, something potentially beneficial for healthcare (Seifert & Seifert, 2019; Baucke et al., 2021). By prioritizing the engagement of stakeholders, such as patients, and emphasizing person-centered and evidence-based practices, collaborative inquiry creates possibilities for coordinated healthcare involving different healthcare professionals. This practice generates relevant outcomes both for stakeholders and health practitioners, such as “promoting relevant knowledge and skills, seeking information, looking for help, engaging in healthful thoughts and behaviors, working for prevention, finding mutually agreeable treatment plans, and achieving competent health management” (Seifert et al., 2019, p.1773).

In mental health, we use the term *co-production* to describe the engagement of patients as a more extreme form of involvement, where the power imbalances between medical professionals and patients are retuned. Co-production can be defined as a process based on an equal and reciprocal relationship between professionals, service users, family members, and community members (Whitley et al., 2019). It involves the active participation of users in all the stages of the program, including design, implementation, and evaluation. Additionally, it does not rely on a single definition of appropriateness and is not limited to a specific group of users selected based on diagnostic or operational criteria (Happell et al., 2018). This process implies that service design, implementation, and evaluation are carried out by a group that is composed of professionals from the mental health disciplines and individuals with direct experience of mental distress (Realpe & Wallace, 2010).

Research shows that co-produced recovery processes in mental health services with users promote identity reconstruction, beyond the labeled social role of the mentally ill, with a resulting significant enhancement of social roles and deep relationships with the community (Rose & Beresford, 2018); prevent the emergence of more acute needs by filling gaps in services and supporting mental health crises (Slay & Stephens, 2013); reduce hospitalization and medicalization, suggesting savings in health expenditure (Boyle & Harris, 2009). These processes also challenge the power asymmetries that are inherent to the medical perspective and promote new and unexpected narratives of subjectivity. Various co-production experiences have been implemented in Italy, from north to south, including in cities such as Brescia, Trieste, and Latiano (Pocobello et al., 2020; Gheduzzi et al., 2019; Sangiorgi et al., 2020). These experiences vary in their scale, level of integration with public mental health services, processes, and /or human and economic) resources employed, and target populations (D’Avanzo & Vallarino, 2016; Pocobello, 2014). Their paucity shows that, to date, psychiatric services, regardless of economic and professional resources allocated to the mental health system, have not yet realized the complete community shift of mental health practices (Saraceno, 2017) and have failed to sufficiently incorporate community development work and promote the structured involvement of users in the programming, production, and evaluation of interventions as a crucial aspect of effective recovery-oriented work (D’Avanzo & Vallarino, 2016). The current literature indicates that co-produced activities can be effective means of change for services and communities (Slay & Stephens, 2013), even if more research is needed.

## Peer Support Workers and Empowering Processes

The examination of co-production has been linked to *peer support work* as a factor in promoting organizational change in a recovery-oriented approach (Repper et al., 2013). Peer support workers leverage their first-hand experience with mental health challenges to assist others and their families in receiving mental health services. They collaborate with other care team members to contribute to their overall well-being and serve as a source of motivation in their journey toward recovery. The Implementing Recovery through Organizational Change (IMROC) platform, developed by Repper and Carter (2011), has collected multiple reflections on the professional formalization of peer support work as a tool for realizing effective forms of co-production. Due to the varied range of roles, forms, and application contexts of peer support workers, IMROC has greatly emphasized the core values of peer support workers in social care organizations, such as the recognition of paid work for individuals with experience of mental illness and their role in a dimension of the creative and sensitive invention (Repper & Carter, 2011). Among the experiences of formalizing peer support workers, IMROC identifies various models of role definition (working in ad-hoc groups or being included in existing working groups) and different forms of peer support that can be institutionally enhanced: (a) natural form, that is the informal network of a user, a user group, or a service segment; (b) participation in specific paths managed by users, which are developed alongside the official services; (c) professional integration in institutional services, directly or indirectly employed. Additionally, IMROC suggests considering variables such as the context of the work (individual or group), the opportunity for decision-making in defining the relationship between the peer worker and the user, and the positioning of the subjects involved at different points in their recovery path when defining the scope of peer support. Peer support workers often use recovery-oriented language and practices that focus on strengths, hope, and resilience; they can act as advocates for people, helping them navigate the mental health system and access the services and resources they need. In general, principles such as mutualism, reciprocity, lack of directivity, focus on resources, inclusiveness, and “safeness” are identified as essential for peer support work, with a particular emphasis on the redistribution of power, enhancement of different forms of knowledge, and sensitivity to specific local contexts (Repper & Carter, 2011).

In community psychology, empowerment focuses on mastery and personal or collective power and refers to self-determination and meaningful connectedness within community life. It is widely recognized as a key principle for orienting mental health services and interventions (Sacchetto et al., 2016). Thus, an approach that focuses on strengths, hope, and a sense of ownership is empowering as it recognizes and builds upon the individual’s capabilities and resources (strengths-based approach) and not only on weaknesses and limitations (deficit-based approach).

Empowerment and strengths-based approach are theoretically coherent and interconnected with the capabilities’ perspective (Corrigan et al., 2006), which provides guidelines for rethinking the role of consumers, restoring their agency and control over their lives (Farkas et al., 2005), as well as their right to choose within socially valued opportunities for integration and citizenship (Nussbaum, 2011).

Empowerment develops through the promotion of agency and responsibility of different actors (people with first-hand experience of mental suffering, professionals, and citizens) in the mental health field and interacting effectively with health services and becoming active partners in managing their illnesses (Wallerstein, 2006). Various studies have highlighted the significance of empowerment in improving health outcomes (see Schneider-Kamp & Askegaard, 2021) and enhancing the overall well-being of individuals (Cattaneo & Chapman, 2010; Grealish et al., 2017). The empowerment model is based on the promotion of processes and outcomes at the individual or psychological level, at the organizational or interpersonal level, and the community or social level (Perkins & Zimmerman, 1995; Zimmerman, 2000). The first dimension includes the processes that can be promoted regarding abilities and skills to make decisions about their lives, expand their capabilities, make informed choices, and work with relevant others. In terms of outcomes, the focus is on raising awareness of the own situation, a perceived sense of control, and behaviors of participation in the community. The second dimension explores the processes of organizational decision-making, shared leadership and responsibility, and the outcomes of organizational development, networking, and influence in policies. The third dimension includes the processes of collective actions to access resources and improve tolerance for diversity and the effects of constructing coalitions and developing pluralism in leadership, and community resources.

This study aims to evaluate the impact of a co-production program run by a community organization on the recovery of its participants. The program emphasizes the importance of peer support workers, who were fully involved in implementing the program and evaluating its effectiveness. The program’s implementation and evaluation were guided by the principles of co-production. Therefore, two main research questions are posited.

RQ1: What are the empowerment outcomes of the co-production process?

RQ2: How do peer support workers promote empowering processes?

## The Context

The organization *L'Arco*-*Corrispondenze per la recovery* (*The Arch-Correspondences for Recovery*, hereafter referred to as *the organization*) is a non-profit organization established in 2017 in Bologna, Italy, by a retired psychiatrist, a psychologist, an educator, and five individuals with direct experience in mental health. The organization’s mission is to promote a recovery process for people with mental health diseases. The organization is composed of four experts with professional qualifications (a psychiatrist, two psychologists, and one educator) and four experts by experience (trained through experiences in user and family associations or through training courses offered by the Department of Mental Health of Bologna as “Experts in Peer Support”). Three facilitators are committed to the organization on a volunteer basis, while five have permanent employment contracts (part-time between 12 and 38 h per week). The organization also has an employee with administrative functions, as well as a business accountant and a labor consultant. This organizational structure aims to ensure competence and stability while promoting the voluntary participation of citizens, people receiving treatment for mental disorders, and their families. Since its establishment, the organization has developed a variety of activities and services for mental health recovery and has reached 312 individuals (as of October 2021) who have participated in individual programs, group activities, and courses. Individual programs (206 related to people who come from mental health services, 55 related to people followed by territorial social services) are based on personal meetings that utilize a three-way co-produced methodology involving two facilitators (one expert by competence and one peer support worker) and the individual seeking support. Individual programs are suggested routes to support and guide the recovery journey, tailored to meet different needs. They are organized flexibly, to find the most appropriate approach in collaboration with the individual. Participants in the organization’s activities do not identify themselves as patients, users, or consumers of mental health services, thus related to the fact that the organization does not describe its practice as therapy or psychotherapy. During the research, we never used the terms “patient,” “user,” or “consumer,” but only the word “participant” to refer to people participating in the organization’s activities and in the research.

Group activities and courses are aimed at raising awareness and training the entire community on mental health recovery-related topics (for example, diagnosis, medications, emotional management, crisis management, etc.) and are co-constructed with participants.

The organization’s intervention is “designed” for people subjectively interested in a path to recovery rather than specific groups identified by a diagnosis. The organization’s activities access is based on voluntary motivations and not linked to the institutional services of the city, although a partnership has been maintained.

## Materials and Methods

### Procedures

A co-produced and circular evaluation process was designed and implemented by an Evaluation Research Team (ERT) composed of two researchers, one sociologist and one psychologist, and six organization members (four of them were peer support workers and two were a psychologist and an educator) with varying levels of competence and experience expertise. The ERT developed the research process through a series of meetings spanning from January 2021 to May 2022 (Table 1).

**Table 1:** Evaluation process phases

Phase 1	1. Collaborative literature review 2. ERT reflection meeting #1: Sharing of archive documents, methods, indicators, and areas to be investigated
Phase 2	3. Selection of participants for focus groups 4. Focus group discussions 5. ERT reflection meeting#2: Preliminary analysis of qualitative emerging data
Phase 3	6. ERT reflection meeting#3: Selection of participants for individual interviews 7. Individual interviews 8. ERT reflection meeting#4: Discussion of preliminary data from focus groups and interviews 9. ERT reflection meeting#5: Final and integrated data analysis, final event preparation 10. Restitution event for the community

The process began with a collaborative literature review on mental health recovery and co-production, which aimed to critically analyze the different implementations and meanings of recovery in existing experiences. Then, the ERT held a reflective meeting in which participants discussed and agreed on the value of the evaluation process for the organization and community, the accessible archive documents, the indicators, and the areas to be investigated. The qualitative design was considered the best evaluation method for the topic of investigation, and participants were included to give them a voice and open the discussions collectively.

In the second phase, the ERT discussed and prepared the focus group grid and inclusive criteria of participants. Four focus group discussions were conducted during spring 2021, followed by a second reflective meeting to analyze the preliminary results. In the third phase, the ERT discussed and prepared the individual interviews grid, with a particular emphasis on choosing effective inclusive criteria for participant selection. The interviews were conducted by one researcher during the winter of 2021. The last two reflective meetings were used to discuss preliminary data from the interviews and to integrate the qualitative results from both the focus group and individual interviews.

The final evaluation meeting, to discuss the integrated results of the evaluation, was conceived as a public event, open to the organization community, mental health services professionals, policymakers, and interested citizens. It aimed to show and discuss the evaluation process results and open a debate in the broad community on relevant issues such as recovery, co-production, and peer support work. Informed consent was obtained from all participants in the study, and measures were taken to maintain confidentiality and anonymity. The procedures adhered to the ethical standards set by the Italian Psychological Association and the 1964 Helsinki Declaration.

### Participants

The ERT members prepared a call for participation in the focus group discussion phase of the evaluation research. Potential participants were contacted by phone and provided with a brief explanation of the research objectives before being asked if they were interested in being involved in the research. A total of 45 individuals, including users of social and mental health services, caregivers or family members, and volunteers of the organization participated in four focus group discussions. The inclusion criteria were (1) having attended activities of the organization (either individual or group activities) during its life course and (2) being available for a collective discussion. The goal was to keep the involvement in focus groups as broad as possible to allow all interested individuals to contribute to this research phase.

For the individual interview phase, 18 persons were selected by the facilitators of the organization. Out of these 18 individuals, 13 agreed to be interviewed (8 cis-gender women and 5 cis-gender men, M_age_ = 47.7, range 27–60 years).

The selection of the 13 people was carried out reflecting on the features of the individual paths and the recovery outcomes. In a reflective meeting of the evaluation research group, we decided to look at the notes the facilitators (according to and encouraged by the facilitators, having previously received their consent) collected weekly to keep track of the individual paths, and we noticed that there were: (a) differences in the duration of individual paths that could vary from a minimum of 5 months to a maximum of 3 years; (b) differences in the recovery outcomes (in terms of perception of satisfaction and/or achievement of goals on the axes of home/work/social inclusion); (c) differences in the recovery process (in terms of pursuing specific objectives home, work, and social inclusion axes and/or attending the organization without a specific focus).

Finally, people were selected according to (1) the duration of individual paths; (2) the perceived and declared satisfaction, and (3) the current condition on the home/work/social inclusion axes.

### Instruments

The focus groups were conducted to collect diverse perspectives on the organization’s processes and activities and the semi-structured interviews were conducted to gain insight into specific recovery stories and personal journeys. Focus groups and interviews were recorded and transcribed, with the previous informed consent of participants.

The focus group guide covered the recovery path and programs promoted by the organization and the participant’s perceptions of their position within these processes. The guide also covered the nature of the co-production process in the organization’s approach, the role of the facilitator in the recovery process, the meanings and values given by people who participate in the organization, and the relationship with the community. The individual interviews aimed to explore the individuals’ journey of recovery, including how the recovery process began, initial perceptions of the crisis, the first contact with the service, the management of the disease, the support received, the most important factors, and the individuals’ role in the process, as well as significant and “turning point” events in the recovery.

### Data Analysis

Data collection and analysis were conducted using qualitative methods, specifically reflective thematic analysis (Braun & Clarke, 2019), to explore the personal meaning of the participant’’ experiences. The data collected through focus group discussions and individual interviews were transcribed and analyzed using a recursive process. To ensure the trustworthiness of the analysis and results, we adhered to four criteria: credibility, transferability, dependability, and confirmability (Guba, 1981).

The transcriptions of each focus group were coded into 37 themes that were synthesized in 5 areas (description of the organization, personal recovery journey in the organization, characteristics of the population, characteristics of the outcomes, relationship with the community) and assigned each segment of the transcription a code. In a reflective meeting, the emerging themes were presented to the entire research group to verify if the group shared and agreed on the salience of the identified themes (credibility). The agreement for each statement was carried out through a progressive qualitative scale with 5 response options (from totally disagree to fully agree). Those with greater convergence were found in the macro areas concerning the description of the organization and the individual journey in the organization. At the end of the 4 focus groups, we co-designed the interviews based on the coding of the focus groups. We decided to focus on the two thematic areas that explore how the organization is described and the processes that occur, dividing them into three major themes: raising self-awareness on the recovery journey, organizational development, and enhancing collective recovery actions.

The whole research procedure was detailed in a diary to ensure transferability and dependability. The two researchers independently coded the data in different stages (dependability). Memos were taken throughout the process, and discussions were encouraged within the team and with the members of the organization to enhance awareness and reflect on any biases, perspectives, and positionality (confirmability).

### Positionality Statement

The authors considered their positionalities reflecting on how their roles and disciplines (two postdoctoral researchers in psychology, an independent researcher in sociology, a tenured professor, and practitioners of the organization), socioeconomic and health backgrounds, and role in the evaluation may have influenced their relationship with the participants. The researchers have never had a personal link but a professional relationship with mental health services and organizations. During the evaluation research, researchers adapted questions and language to the participants’ words, definitions, and identifications and encouraged them to express any doubts, comments, and criticism to improve the following interviews. A reflexive and iterative method was developed to challenge the imbalance of powers between the two professional researchers, professionals of mental health, and peer workers involved in the research (Rivera-Segarra et al., 2022).

## Results

The analysis performed on the data collected resulted in the identification of six codes, which were subsequently organized into three main themes (Table 2). Quotations from focus group discussions are presented according to the focus group in which participants were involved (for example, FG1); for interviews, a code is assigned to each interview (for example, I5).

**Table 2:** Themes and codes from the thematic analysis

Theme	Codes	Description of the theme and codes
Raising self-awareness on the recovery journey	Exploring mental health disease and recovery meanings Redefining identity	The theme explores the individual effects of joining the organization recovery paths in terms of raising awareness on the disease, on personal strengths and on the representation of self (intra-personal dimension of empowerment)
Organizational development	Recognizing peers as co-workers Valuing organization as a safe place	The theme analyses the empowering processes implemented by and in the organization focused on the involvement of peer in the co-production and the development of an organization climate and culture of safeness. (inter-personal dimension of empowerment)
Enhancing collective recovery actions	Framing recovery networks Building a community	The theme analyses the empowering processes related to the promote the recovery approach in the local community, by strengthening existing networks.

## Raising Self-Awareness on the Recovery Journey

This theme shows the personal meanings and representations of recovery, considered an individual process. Every participant focused on individual aims, coping strategies, and effects of mental health recovery.

### Exploring Mental Health Disease and Recovery Meanings

The participants in this study reported on the process they faced in giving a new meaning to their recovery. It was noted that some participants realized that the words related to recovery and mental illness, which are commonly used in diagnostic manuals and by mental health professionals, changed their meaning for them. A relevant quote from one participant highlighted the importance of the origin and context-related definition of mental illness in determining the representation of people suffering from it. This awareness could be considered an important step in redefining the concepts of illness/health and normality.

In analyzing mental illness that is historically and socially determined, I believe the classification of mental illness is a medical classification to manage divergent thinking and behavior. What is not considered canonical at the social level is classified as abnormal, a socially determined category. And the myth of medical treatment is built accordingly. […] it does not exist in a vacuum, as we are inscribed in society and must live with it, but we must relativize the concept of mental health to understand it and cope with it. (FG3)

A certain representation of illness comes with a representation of care that is managed with pharmaceutical treatments instead of relying on the bio-psycho-socio-political representation of the person.

They told me that I had to take the medicines forever because doctors attributed the cause of the disease to chemical decompensation in my brain. In the [recovery] journey, I learned that much depends on the type of life you live. [I9]

Recovery assumes a different meaning, thus being a learning empowering process, a way to train knowledge, competences, and cultural values. In this process, the innovation is presented as a redistribution of roles (peer support workers and professors) and a sharing of knowledge (from discipline and direct experience) that creates a collaborative context in which a sense of hope is envisioned.

There was a process of sharing and transmitting culture, an interesting moment of study, which, for me, was the point of access to the experience of the organization. This in-depth path has allowed us to see mental health service users in different ways/roles than the usual ones. Seeing a user explaining a complex concept with a professor, hearing about how users could work in services, and collaborating with mental health service workers, was very important. We have seen an innovative reality carrying on the discourse of recovery, resuming the thread of one’s existence, different from the approach used in services. Here, this hope remained and was realized. [I7]

Recovery is considered a co-construction process of knowledge, but some participants, struggle to communicate this meaning with individuals within the service sector effectively and to raise awareness regarding the importance of considering the person as a holistic entity. However, during this process, individuals may commonly feel isolated, especially when professionals believe that recovery is not possible and mental illness cannot be cured, even if the individual reports feeling like they are in the process of recovery.

My doctor now says that I am “untreatable”. Perhaps he means I am not sick anymore and does not understand why I am still in contact with the service. [I7]

The recovery process is conceived by the participants as affecting their personal lives in exploring some strengths (and weaknesses) that lead to developing self-confidence, a sense of ownership, and autonomy.

The organization was helpful because I had a life of immobility. Instead, the movement within the organization helped me. I felt more confident. (FG3)

The participants in this study demonstrate an awareness of the strengths that have been developed with the support of the organization’s activities, such as “the movement” and “people around you” which foster meaningful and trustworthy relationships. These aspects aid individuals in identifying their path to recovery, emphasizing the importance of understanding one’s strengths and weaknesses and learning how to manage them effectively. Additionally, even during the most acute phases of suffering, the perspective is re-framed to focus on personal assets and strengths as a means of coping with “the hard times”.

I was hospitalized quite frequently during the crisis, but here I seem to have learned the possibility of another way when there is a crisis. To find a system to be able to get out with my strengths without hospitalization. [I10]

### Redefining Identity

Participants reported that accessing the organization and its activities resulted in a redefinition of their sense of self, as they felt respected, welcomed, and treated with dignity. The redefinition of identity was particularly reported compared to the lack of this opportunity in the public mental health services that often rely on the identity of the ill or patient.

In other places, you are defined by your illness and seen as a problem rather than a person. Here, however, you are considered a person and given the opportunity to talk to someone, rather than just being defined by your illness. (FG2)

The organization utilizes an approach and methods that are rooted in the principle of collaborative listening, whereby the unique experiences of each individual are attentively heard and considered. Furthermore, this approach is informed by the understanding that each person must be viewed holistically, including the presence and impact of their mental illness.

## Organizational Development

The theme of power representation and distribution within the organization and in the process of co-production is here examined as a feature of organizational development.

### Recognizing Peers as Co-Workers

The participants emphasized the significance of peers in the co-production process and the recovery journey. They noted that the knowledge and experience of peer support workers serve as a foundation for navigating power dynamics in the recovery process and for fostering a sense of personal agency. Many participants also acknowledged the value of a three-way approach (professional, peer support worker, and individual seeking support) as a facilitating factor.

If I think about the organization, it helped me more, because it is different from other services. Having an experienced user at the meetings was greatly helpful. [I5]

The strength of the individual’s experience is augmented by the support of a professional and an expert who has personal experience of suffering from a mental disorder. This trio of support is not only characterized by a balanced relationship but also by a shared understanding of the experience. This combination is a winning formula. (FG1)

Others view the proactive role of people as a crucial component in progressing through the recovery journey and in acquiring a sense of ownership. This perspective is grounded in the belief that providing individuals with the opportunity to make choices, actively engage in the process, and take responsibility for their recovery can significantly contribute to their progress.

In my opinion, [the organization] fosters an attitude of responsibility. As a result, one moves from a passive patient to someone who takes ownership of their recovery path. One is no longer just someone who is required to take medication, visit specific places, or perform certain actions, but rather someone who makes conscious choices to follow that path because they understand the reasoning behind it. [I10]

You are not just someone who is required to do things, but rather someone who actively chooses to engage in certain actions. (FG2)

### Valuing Organization as a Safe Place

The participants revealed that the structure and functioning of the organization, including the methodology adopted and the role of peer support workers, provides the opportunity to create a safe environment in which labeling, stigmatization, and prejudices are minimized, and inclusive processes are promoted. This can have a positive impact on the recovery journey of individuals seeking support and can foster a more holistic and respectful approach to mental health care.

Thanks to the presence of both professionals and peers, the organization is a safe environment. Here, you are not just reduced to a psychoanalytic or psychiatric label. They don’t impose a specific approach on you; you have a say in your recovery journey. (FG2)

Gaining this awareness can be considered the foundational step for autonomy and ownership of the recovery process. This process is not only initiated by this awareness, but it is also advanced through the implementation of a co-production approach within the organization. This approach shapes the organizational climate and the interpersonal relationships within the organization, thus creating an environment that is conducive to recovery, one that can be considered “a safe place” where individuals are encouraged to freely express their subjective experiences without fear of judgment or stigmatization. In this sense, a critical perspective on the diagnostic labels is reported by participants, considering the positive side of sharing a clinical label and the related emotional consequences with a peer, who knows, by experience, the suffering moments in life and can share his/her emotional background.

It is an environment where I feel free to express my strange experiences, and my symptoms, especially those that are less common and difficult to deal with elsewhere. [I6]

it’s a safe and free place. I found it to be a haven not because it protects me, but because I am sure I can be completely myself here. We talk together; it matters that those who talk to you also have a diagnosis. There is a different kind of empathy. (FG4)

To participants’ account, the sense of safety entails the ability to express one’s identity and subjectivity while also feeling both personally and socially included, and not discriminated against for suffering from a mental health disorder. The positive experience is rooted in the sense of belonging and acceptance that is offered by the organization. The significance of feeling understood and accepted is paramount as it is a vital component of peer support which is highly effective in promoting recovery. Furthermore, the shared understanding that arises from this sense of belonging and acceptance leads to a unique form of empathy.

## Enhancing Collective Recovery Actions

This theme is related to the promotion of recovery at the community level, by recognizing the value of formal and informal networks and constructing community bonds.

### Framing Recovery Networks

In the recovery process and within the system of mental health services and recovery-oriented initiatives, the organization does not stand that it is not an “alternative” to institutional services but aims to provide a space in which to carry out a path of individual growth that can also improve the relationship between the participant and institutional services (as reported by the organization statute available online). Indeed, the types of services offered, and the methodology adopted by the organization does not overlap and it is not interchangeable with public and private mental health services. The difference with the organization is substantial but in a complementary perspective. The co-production approach promoted by the organization is still, if not more, useful to understand the difference with the other existing services because less affected by the projective logic of conflict with other services.

It offers different support from both services and psychotherapy. It is not interchangeable. I did all three together and they worked. (FG2)

We must consider that we do the first access to the services, at the time of the onset of the malaise, because I doubt that people who have not already been followed by the services, can have access here. (FG3)

It is crucial the awareness of this complementarity and the need to not compare the organization with public services. Indeed,

Making a comparison with services is an extreme and unfair comparison, they have different purposes. The public service has to guarantee the basic functions of an individual, you have to sleep, you do not have to decompensate, and check at the social level that you do not hurt yourself and others. While the aim of the organization is empowerment. (FG3)

### Building a Community

In this code, we have compiled the participants’ references to the concept of community, referring to the effort and commitment of the organization with the broader community of organizations and public services, thus constructing partnerships and building a network for mental health recovery.

This is a small community for exchanging relationships, and there is a need for a wider community: more associations like this could network to support this phenomenon […]. There is also hope for a more welcoming community. (FG1)

Implement the network to ensure that the organization serves as the hub for connecting with cultural and work opportunities, support for social and work inclusion, and as an association that functions as a gateway to the world. (FG1)

The necessity for a network that advocates for recovery across various domains of life (such as social inclusion in the workplace and cultural contexts) is emphasized, and the organization is perceived as a means of creating opportunities for improvement in one’s life.

## Discussion

Our research aimed to investigate the co-production process in mental health recovery within a community organization in Italy. We employed a qualitative evaluation design to gain a deeper understanding of empowering outcomes and processes in the recovery journeys. The organization and its experience can be situated within the context of self-organized action by civil society (Realpe & Wallace, 2010) and offer a critical perspective on the wide range of mental health activities.

Regarding our first research question about empowerment outcomes at the individual level, our findings indicate that the recovery experience is a personal journey. During this process, individuals may not always have clearly defined goals and objectives. Participants are constantly involved in a learning process to acquire and develop strengths and assets to manage their recovery, such as their sense of ownership and self-efficacy (Topor et al., 2011). This suggests that the path to empowerment and mental health recovery varies from person to person, and it might not always follow a linear or standardized trajectory. The journey is essential because it is not predetermined by an existing a priori model, but rather is a co-produced model informed by the values of identity, dignity, and self-stigmatization (Pocobello et al., 2020). Empowerment outcomes include the opportunities for social engagement provided by the organization, such as group and course activities, and peer support, which can help individuals in building connections with others who understand their experiences and foster a sense of belonging (WHO, 2021). The co-production process promotes meaningful support and personal and collective resources, by guiding them to set realistic goals, make positive changes, and cope with setbacks. Furthermore, it promotes an active role of people in their recovery by involving them in the co-production process, listening to their needs and preferences, and providing them with the tools and resources they need to achieve their goals (Carpenter & Raj, 2012; SISM, 2021).

Regarding our second research question, the role of peer support workers in the recovery process is fundamental. This is due to the organization’s strong emphasis on peers, involving them in the collaborative design of activities. Peers actively work with individuals, their families, and community members to co-create and co-design mental health activities and programs that cater to the unique needs and preferences of the community. Their involvement ensures that the initiatives are more responsive and relevant to the diverse range of individuals they serve (Mancini, 2018). In addition, peers are also engaged in a collaborative evaluation of individual paths to assess their effectiveness and make necessary changes. Peers provide a unique form of empathy and understanding, which can be particularly valuable for those who are in the early or late stages of recovery. They can tailor their approach to individual preferences and can support participants to feel at ease and open to trying the resource in the first place (Li et al., 2022). They also serve as advocates for their peers and work to create an inclusive and supportive environment within the organization (Kemp & Henderson, 2012). Peers’ support enhances the construction of a sense of community, creating both a feeling of emotional connection that facilitates the sharing of struggles and mental health management strategies, and a connection to the broader community through meaningful and trustworthy relationships. This process allows people to define themselves as full individuals with mental health conditions in a more proactive way.

## Conclusions

The results of this study emphasize the importance of community support for mental health recovery and the power of peer support, coherently with the empowerment model in the community psychology approach. This research shows the relevance of involving individuals and communities in the co-production of mental health recovery within community organizations. Engaging community members and involving them in the design, implementation, and evaluation of mental health programs can ensure that the services provided are responsive to individuals and community’s specific needs and are more likely to be adopted and sustained over time. Our study provides some evidence of the contribution of co-production to the recovery process and can serve to promote a culture of co-construction in mental health services, where community organizations can contribute to their transformation. The emergence of unresolved issues in research (outcomes and their measurement, the ability to describe the population’s characteristics compared to the overall population accessing health and social services, and how to define the relationship with the community) suggests the possibility of further interaction with institutional services. In this direction, people who choose to participate in community organizations, create a bridge (or an arch) to make the actions of public institutions closer to the existential events of the population they serve. Finally, it seems crucial to develop and enact policies that provide peer workers and community organizations with allocating time, funding, and autonomy to use innovative recovery-based resources to improve mental health systems. Rather than providing a one-size-fits-all model, the deepest formalizations of recovery processes in literature have valued the variability of the personal paths that can develop, connected to the multiplicity of possible organizational formulas. Our results reveal that qualitative description can offer an in-depth understanding of the personal paths and links between co-production, a sense of “safety” and “being taken seriously”, and the reconstruction of the self. As WHO (2021) noticed, a large group of people suffer from mental distress but cannot find their needs met because of poorly organized services, with neglect of human rights and stigma that operate as a difficult threshold to access. The organization involved in this study, provides an empowering context to redefine the meanings of patient-centered perspectives. As defined by Schneider-Kamp and Askegaard (2021), the organization is a community organization where psychotherapists and non-professionals are peers, which can be considered an empowerment initiative not based on a medically centered perspective. Therefore, the organization is not an empowerment initiative that begins from a medical authority institution but rather a community-based initiative. The organization’s specific feature is being a community organization that promotes recovery based on individual choice and adherence of participants, thus presenting forms of agency that cannot be attributed to medical-centered models.

By delving deeper into the connection between co-production, recovery, and the reconstruction of the self, it becomes possible to envision organizations that bridge the gap between needs and responses. The position of the organization within the network of mental health community organizations and its relationship with public mental health services is peculiar. As an organization based on activism and community work, it can catalyze further innovations in public services. Recovery-oriented care assumes that users and professionals collaborate toward achieving the goals defined by the user. However, despite the emphasis on patient-centered or consumer-oriented care, service delivery decisions often remain controlled by providers and administrators. This disparity can lead to a lack of genuine partnership and empowerment for the individuals seeking support. Mere involvement of users in treatment planning does not guarantee a partnership. A significant shift in the balance of power and control is necessary to establish genuinely collaborative approach.

This study is not without limitations. The research evaluation design cannot provide a comprehensive understanding of the general mental health services population, as our results are specific to the context of the community organization involved in the study. The study focuses on specific co-production processes developed within a community organization that is part of a network of organizations dealing with mental health activities and services. Additionally, the time-consuming and resource-intensive nature of collaborative evaluation research meant that not all individuals accessing the organization were able to participate and contribute.

Despite the limitations, the study offers a clear picture of how co-production can be structured in a community-based mental health organization, shedding light on the processes that drive, support, and nurture the recovery process, above all promoting empowerment and having an active role in decision-making processes.

## Data Availability

The datasets generated during and/or analyzed during the current study are available from the corresponding author upon reasonable request.
